# Physician assignment of causes of death from verbal autopsies

**DOI:** 10.2471/BLT.26.295870

**Published:** 2026-09-01

**Authors:** Chalapati Rao, Henry D Kalter, Daniel Chandramohan, Daniel Cobos, Jordana Leitao

**Affiliations:** a National Centre for Epidemiology and Population Health, Australian National University, 62, Mills Road Acton ACT 2601, Australia.; b Department of International Health, Johns Hopkins Bloomberg School of Public Health, Baltimore, United States of America.; c Department of Disease Control, London School of Hygiene and Tropical Medicine, London, England.; d Epidemiology and Public Health, Swiss Tropical and Public Health Group, Basel, Switzerland.; e Lisbon, Portugal.

Information on causes of death in a population is essential for evidence-based health actions, policy development and evaluation, and epidemiological research.^1^ While medical certification of causes of death is the optimal source of such data, many deaths in low- and middle-income countries occur outside medical attention, which limits data availability.^2^ To address this gap, the World Health Organization (WHO) recommends verbal autopsy methods, which involve using a standard questionnaire and methods to collect information from the family of the deceased on the symptoms, signs and events related to the terminal illness.^3^^,^^4^ The verbal autopsy data are subsequently analysed to derive the cause(s) of death, either through the conventional approach that uses physician reviewers or through the more recent development of computerized diagnostic systems.

In the last three decades, concern regarding the potential limitations of verbal autopsy diagnoses has increased because these diagnoses rely on personal clinical judgement, which could vary across practitioners. To some extent, this concern is driven by inherent variations in verbal autopsy data arising from inconsistent reporting or recording of information in the open narrative, structured questions or transcription of available medical records in different sections of the verbal autopsy questionnaire. However, a trained physician could decipher the logic of the case by sifting through the responses and evaluating abnormal or inconsistent information, while recognizing the reliability of certain elements of evidence in arriving at the probable diagnoses.^5^ Nevertheless, such interpretation is essentially based on personal clinical judgment, hence the potential for bias.

## Physician reviewers’ manual

To enable a standardized approach and improve the consistency and comparability of physician-derived verbal autopsy causes of death, the WHO Verbal Autopsy Reference Group has developed a detailed manual to guide physician reviewers in their tasks.^6^ The manual includes several chapters that cover the background principles, need for, uses and methods of verbal autopsy data collection. A chapter covers the basic principles of medical certification of causes of death and procedures for selecting the underlying cause for each death. This chapter includes basic descriptions of the parallels between verbal autopsy-based information on illness signs, symptoms and clinical events and related medical diagnoses of causes of death. The manual also includes diagnostic guidelines for the WHO list of common causes of death that can be identified from information recorded at verbal autopsy interviews, and is to be used by physician reviewers to increase the objectivity and reliability of verbal autopsy diagnoses. The final chapter provides guidance on training programmes for physicians in performing their tasks, including details of topics to be covered in specific training sessions, along with exercises for assessing consistency of cause of death assignment practices and reliability of verbal autopsy diagnoses across participants. The manual is a major step towards improving the quality and reliability of verbal autopsy-based causes of death assigned by physician reviewers, and the resultant utility of the data.

## Alternative approaches 

Several research groups have independently developed diagnostic software as an alternative approach to deriving the probable cause of death from verbal autopsy data.^7^ From an operational standpoint, this approach has several advantages in terms of implementation efficiency and potential for consistency of outcomes, because there is no need to arrange for a physician to review the verbal autopsy data, incorporate the diagnoses into the data set and undertake other related activities. Limited availability of field staff as well as financial resources for verbal autopsy implementation have also contributed to the increased interest in diagnostic software as well as the use of artificial intelligence (AI) for deriving causes of death from verbal autopsy. A recent analysis that compared the performance of AI models and diagnostic software with physician coding of verbal autopsy provided foundational evidence for integrating these innovations with physician review to improve the quality of verbal autopsy data.^8^ Physician review and computer-coded verbal autopsies are needed, separately or combined, depending on the resource settings. The international recommendation is that physician review should be used at least in a sample of cases in the reference population, to calibrate the quality of verbal autopsy diagnoses generated by diagnostic software.^7^

## Physician verbal autopsy benefits

To meet the need for causes-of-death data, the international public health community is increasingly focusing on introducing and scaling up verbal autopsy data collection in countries where death registration systems have gaps in medical certification. In several countries, verbal autopsy is necessary when the death occurred at home without medical attention and hence could not be medically certified. However, for many such deaths, reasonably adequate clinical information for the deceased might be available from the household, in the form of recent hospital discharge notes, medical prescriptions, or laboratory or imaging reports and referral letters, among others. During the interview, such medical information is requested and recorded in the open narrative or medical records section of the questionnaire.^9^ Physicians reviewing the verbal autopsy can collate this information with the structured responses to determine the causal chain as well as identify contributory conditions. Doing so enables the application of coding rules to select the appropriate underlying cause of death, while also identifying multiple causes, which are becoming increasingly important to evaluate complex causality particularly in the case of mortality from noncommunicable diseases. When derived from verbal autopsy that applies coding rules, underlying causes are compatible to be merged with those derived from medically certified deaths, through application of the same coding rules. Subsequently, the merged data set could be used to estimate population-level cause-specific mortality indicators. Physician review is also preferred where verbal autopsy is used as part of mortality audit activities, as well as to investigate disease outbreaks or public health emergencies. In addition, findings on verbal autopsy data quality from physician review can be used to enhance the quality of field verbal autopsy data collection.

## Implementation challenges

Despite these advantages, field implementation can be challenging. In some countries, verbal autopsy can be implemented only in sample or sentinel clusters, owing to logistic considerations as well as limited availability of field health workers to conduct household interviews. Recent reductions in official development assistance for health have adversely affected public health surveillance in about 42% (45/108) of low- and middle-income countries, which would also affect verbal autopsy programmes.^10^ Furthermore, in some remote areas physicians are scarce, and some locations have high clinical workloads, which limits the time for verbal autopsy review and cause-of-death assignment. In countries with high clinical staff turnover in rural areas, capacity-building of interviewers as well as physicians is a recurrent need. On average, physician review of a verbal autopsy record takes about 15 minutes, and primary care practitioners in rural areas could absorb three to four reviews each week into their routine workload. Incorporating this function into the routine activities of primary care physicians would need to be supported by adequate training, along with technical assistance for problematic cases.^9^^,^^11^ To overcome challenges in management of paper-based verbal autopsy implementation, national teams have designed tools and processes to enable data collection and physician review in a seamless digital environment, which have substantially enhanced timeliness and efficiency.^12^

All these challenges need to be accounted for by verbal autopsy managers during programme design and when planning case allocation. Expert review is also needed in a representative sample to assess adherence to the diagnostic guidelines for specific causes of death, as well as to assess the overall reliability of physician-certified verbal autopsy diagnoses. Findings from such assessments should be used to improve data collection as well as the utility of derived causes of death. Also, since verbal autopsy diagnoses are based on retrospective household interviews, they can be used for health sector purposes such as policy development, analysis and research but not for legal or other administrative functions.^13^ Governments should specify, in relevant instructions issued at the start of implementation, the specific and limited health-sector uses of verbal autopsy-derived causes of death. Such notification will enable physician reviewers to perform their functions to the best of their knowledge and belief, based on the information presented to them from the verbal autopsy.

## Way forward

Verbal autopsy-based cause-of-death data present inherent uncertainties, despite the attention to quality assurance, control of data collection, standardization of physician cause of death assignment and development of computer-assisted verbal autopsy diagnostics. Verbal autopsy is at best an interim arrangement while national mortality data systems are being scaled to achieve total coverage of medical certification of causes of death.^14^ However, implementing verbal autopsy in countries with gaps in data availability is necessary to improve national and global vital statistics. In this regard, the implementation of physician review serves as a vital step in improving national capacity for cause of death certification, which is to be implemented by primary care physicians mostly serving rural or remote areas, as well as by those providing community health services in urban areas. Doing so will require integration between death registration and community health information systems to enable timely verbal autopsy investigation of causes for deaths that are registered without a medically certified cause or identified by a population-based surveillance system. Trained and experienced physician reviewers would then need to expand their role to medically certify community deaths within their areas. For some of these deaths, they would already be aware of the clinical histories and/or have access to relevant clinical documentation. Introducing household verbal autopsy interviews by field staff along with physician review functions for assigning causes of death can accelerate the availability and quality of population-based cause-of-death data at district, state or provincial, national, regional and global levels, for evidence-based public health analyses and action.
